# Multi-omics and machine learning-based exploration of key genes associated with abdominal aortic aneurysm

**DOI:** 10.3389/fmolb.2026.1786983

**Published:** 2026-05-29

**Authors:** Ming Xie, Yong Xue, Yufeng Zhang, Lei Zhang, Xiandeng Li, Guobao Chen, Jia Liu, Haibing Hua

**Affiliations:** 1 Department of Pharmacy, Jiangyin Hospital of Traditional Chinese Medicine, Jiangyin Hospital Affiliated to Nanjing University of Chinese Medicine, Jiangyin, China; 2 Department of Cardiology, Jiangyin Hospital of Traditional Chinese Medicine, Jiangyin Hospital Affiliated to Nanjing University of Chinese Medicine, Jiangyin, China; 3 Department of Vascular Surgery, The Second Affiliated Hospital of Shandong First Medical University, Tai’an, China; 4 Postdoctoral Workstation, Shandong First Medical University & Shandong Academy of Medical Sciences, Jinan, China; 5 Department of Pulmonary and Critical Care Medicine, Jiangyin Hospital of Traditional Chinese Medicine, Jiangyin Hospital Affiliated to Nanjing University of Chinese Medicine, Jiangyin, China; 6 Department of Laboratory Medicine, Jiangyin Hospital of Traditional Chinese Medicine, Jiangyin Hospital Affiliated to Nanjing University of Chinese Medicine, Jiangyin, China; 7 College of Pharmacy, Chongqing Medical University, Chongqing, China; 8 Department of Gastroenterology, Jiangyin Hospital of Traditional Chinese Medicine, Jiangyin Hospital Affiliated to Nanjing University of Chinese Medicine, Jiangyin, China

**Keywords:** abdominal aortic aneurysm, biomarker, colocalization, machine learning, Mendelian randomization

## Abstract

**Background:**

Abdominal aortic aneurysm (AAA) represents a high-risk arterial pathology that frequently evolves insidiously and remains without robust molecular tools for timely detection. To identify potential biomarkers with both genetically supported relevance and discriminatory value, we developed an integrated multi-omics framework that synthesizes genetic, transcriptomic, and proteomic evidence.

**Methods:**

Two microarray datasets (GSE47472, GSE57691) were merged following batch correction and validated using GSE7084. To nominate candidate genes and proteins with genetically supported relevance to AAA, we integrated genome-wide expression and protein quantitative trait loci (eQTL and pQTL) summary statistics within a two-sample Mendelian randomization (MR) analytical design. Differentially expressed genes overlapping MR-supported candidates were further refined using multiple complementary machine learning (ML) approaches. Bayesian colocalization was employed to investigate the extent to which gene expression regulators share genetic architecture with AAA susceptibility. Discriminatory performance was evaluated by constructing receiver operating characteristic curves, and expression changes were validated in a calcium chloride-induced murine AAA model through experimental validation.

**Results:**

A set of 551 genes exhibited significant differential expression in AAA tissues relative to non-aneurysmal controls. MR analyses revealed 267 eQTL-supported genes and 129 pQTL-supported proteins associated with AAA risk, yielding 14 overlapping candidates. ML integration consistently prioritized 4 genes—PLAU, CD58, PCYOX1, and THBS4. Among these, PLAU demonstrated strong colocalization with AAA risk loci (PPH_4_ = 0.983) and robust discriminatory accuracy across training and validation cohorts (area under the curve = 0.854 and 0.944). Gene set enrichment analysis and immune-infiltration profiling indicated that elevated PLAU expression is associated with complement activation and enrichment of pro-inflammatory immune subsets, including activated dendritic cells, natural killer T cells, regulatory T cells and T helper 17 cells. *In vivo* validation demonstrated marked elevation of PLAU expression at both transcript and protein levels in aneurysmal aortas, alongside increased circulating PLAU levels in peripheral blood.

**Conclusion:**

This integrative MR-ML-colocalization strategy provides a comprehensive framework for prioritizing potential biomarker genes in AAA. Convergent multi-omics evidence, together with experimental validation, identifies PLAU as a robust candidate gene strongly associated with genetic susceptibility and immune-inflammatory vascular remodeling.

## 1 Introduction

Abdominal aortic aneurysm (AAA) is a progressive and potentially lethal dilation of the abdominal aorta, clinically identified when the vessel enlarges by at least half of its normal caliber or reaches a diameter greater than 3 cm (de Bruin and Verhagen, 2024). Although frequently asymptomatic, AAAs may rupture abruptly, leading to massive internal hemorrhage and very high mortality (90%–100%) (Golledge et al., 2023). Epidemiological studies consistently implicate cigarette smoking, hypertension, advanced age, male sex, and family history as major risk factors; however, these factors are not specific to AAA and overlap with other cardiovascular diseases (Kent, 2014). Early detection is therefore essential for improving outcomes. At present, abdominal ultrasonography is the most widely accepted modality for identifying subclinical AAA owing to its high sensitivity and favorable cost-effectiveness (Force et al., 2019). Nevertheless, imaging alone provides limited information on rupture risk or short-term disease progression, particularly at early stages, underscoring the pressing need for robust molecular indicators capable of supporting disease evaluation and patient stratification, and to inform therapeutic development.

AAA is driven by complex pathophysiological processes, including chronic inflammation, extracellular matrix (ECM) degradation, oxidative stress, and immune cell infiltration (Li and Kong, 2020; Marquez-Sanchez and Koltsova, 2022). To interrogate these mechanisms at the molecular level, transcriptomic studies—predominantly leveraging public resources such as the Gene Expression Omnibus (GEO)—have identified numerous differentially expressed genes (DEGs) in AAA compared with healthy aortic tissues (Xie et al., 2024). Functional enrichment analyses consistently implicate pathways relevant to AAA pathogenesis. However, conventional transcriptome-based investigations are primarily correlational, limiting the discrimination of potential disease drivers from secondary responses. In addition, single-omics designs may not capture the multifactorial nature of AAA, underscoring the need for integrative approaches that more effectively prioritize biologically meaningful targets.

To resolve these issues, Mendelian randomization (MR) serves as a rigorous tool for assessing potential causal relationships between genetic variation and disease susceptibility. MR employs germline variants as instruments, thereby minimizing confounding effects and avoiding the reverse causality issues common in observational research (Davey Smith and Hemani, 2014). When integrated with large-scale expression quantitative trait loci (eQTL) and protein quantitative trait loci (pQTL) datasets, MR enables the mapping of regulatory variants that modulate gene and protein abundance (Ferkingstad et al., 2021; Vosa et al., 2021). This multi-omic MR strategy facilitates cross-validation across genomic, transcriptomic, and proteomic layers, thereby enhancing the specificity and reliability of biologically relevant targets. Accordingly, MR complements DEG-based analyses by providing genetic support for molecular associations in AAA.

Building on these advances, recent studies increasingly adopt multi-omic integrative strategies that combine transcriptomic, genomic, and proteomic data to identify biomarkers with functional and biological relevance (Kreitmaier et al., 2023). These approaches enable cross-validation across molecular layers, thereby improving the robustness of candidate prioritization. However, the substantial complexity and variability inherent in omics datasets hinder the identification of biomarkers with true translational or therapeutic relevance. Machine learning (ML) has become an effective tool for extracting informative features from complex datasets and to construct predictive models for disease classification and risk stratification (Libbrecht and Noble, 2015). Moreover, coupling ML with MR allows biomarker refinement from both data-driven and genetically-informed perspectives. This integrated paradigm strengthens confidence in prioritized targets and deepens biological understanding of AAA pathobiology (Cui et al., 2023).

**FIGURE 1:**
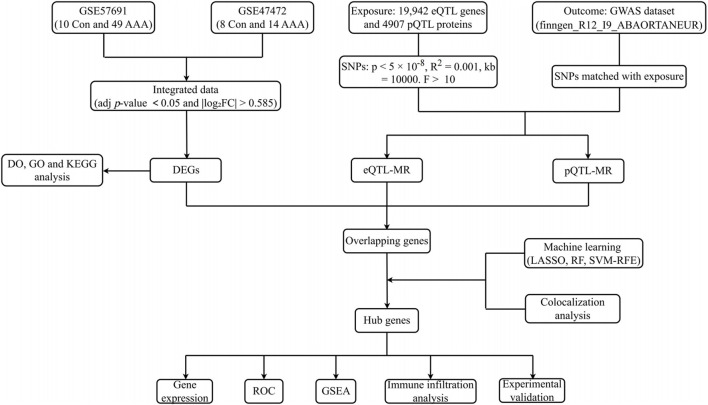
Diagram summarizing the study design.

This work sought to pinpoint biomarkers that are genetically supported, and highly discriminatory for the molecular characterization of AAA. The transcriptomic data were first processed to screen for genes differentially expressed between AAA and control tissues. These DEGs were then integrated with eQTL and pQTL data to connect transcriptional and proteomic alterations to genetic regulation. MR analyses were used to prioritize biomarkers with genetically predicted effects on AAA. To improve candidate ranking and determine discriminatory ability, we developed ML models and assessed their classification performance by generating receiver operating characteristic (ROC) curves. In addition, we employed both gene set enrichment analysis (GSEA) and correlation analyses to investigate the underlying biological processes. To verify the translational significance of our observations, additional experimental validation was performed. By combining multi-omics data with genetic inference, our framework identifies PLAU as a biomarker candidate for AAA. PLAU encodes the urokinase-type plasminogen activator (uPA), a key mediator of ECM remodeling and vascular inflammation, both of which are hallmark processes in aneurysmal progression (Hindy et al., 2022). The study workflow is summarized in Figure 1.

## 2 Materials and methods

### 2.1 Data acquisition

We obtained human AAA-related microarray profiles from GEO (Barrett et al., 2013), applying the criteria outlined below for dataset inclusion: (i) array-based gene expression profiles from human aortic tissue; (ii) presence of both AAA and non-aneurysmal control samples; (iii) availability of accompanying sample-annotation files; and (iv) public availability of both raw and processed data. Three datasets satisfied these criteria—GSE57691, GSE47472, and GSE7084—and were included in the analysis. A summary of their characteristics is provided in Table 1.

**TABLE 1:** Characteristics of the three GEO datasets.

GSE ID	Platform	Tissues	Samples (Con/AAA)	Control source	Attribute
GSE57691	GPL10558	Abdominal aortic	10/49	Organ donors	Training
GSE47472	GPL10558	Proximal neck of abdominal aorta	8/14	Organ donors	Training
GSE7084	GPL570,GPL2507	Abdominal aortic	10/9	Autopsy specimens	Validation

Con: control. AAA: abdominal aortic aneurysm.

### 2.2 Data preprocessing and identification of DEGs

GSE57691, GSE47472, and GSE7084 were imported and preprocessed in R (version 4.3.2). Dataset-level preprocessing and normalization were applied to the raw expression data to mitigate potential batch effects across studies (Leek et al., 2012). GSE57691 and GSE47472 were then merged to form the training cohort, whereas GSE7084 was held out as an independent validation cohort. DEGs were identified with the limma package (Ritchie et al., 2015) with adjusted (adj) *p*-value <0.05 and |log2 fold change (FC)| > 0.585. Volcano plots and heatmaps were generated with pheatmap. For gene-level normalization, we used GEO-provided expression profiles along with their associated platform annotation files. Principal component analysis (PCA) was conducted to evaluate potential batch differences between studies and to visualize sample separation.

### 2.3 MR analysis

#### 2.3.1 Exposure and outcome datasets

Exposure datasets were sourced from the eQTLGen consortium, comprising eQTL profiles generated from 31,684 whole-blood and peripheral blood mononuclear cell samples, covering 19,942 genes (Vosa et al., 2021). PQTL datasets were derived from genome-wide association study (GWAS) summary statistics generated for 4,907 plasma proteins measured across 35,559 participants in an Icelandic population cohort (Ferkingstad et al., 2021). Outcome GWAS information was derived from FinnGen Release 12, offering summary-level genotypes for 467,545 participants of European ancestry, consisting of 4,439 AAA cases and 463,106 control subjects (Kurki et al., 2023). The release can be accessed via the FinnGen website. Regarding reference genomes, the eQTL data used GRCh37 (hg19), while both the pQTL and outcome data used GRCh38 (hg38).

#### 2.3.2 Instrument selection and linkage disequilibrium (LD) clumping

After assembling the eQTL- and pQTL-based exposures, instrumental variables (IVs) were selected through a multi-step procedure. First, single nucleotide polymorphisms (SNPs) showing strong associations with gene or protein expression were selected when reaching the genome-wide significance level of *p* < 5 × 10^−8^. Second, LD clumping was applied to minimise correlation among instruments (*R* ^2^ < 0.001 within a 10,000-kb window). Third, instrument validity was evaluated using *R* ^2^ and the F-statistic, with IVs required to have F > 10 to avoid weak-instrument bias. Given the mixed genome builds, our MR harmonization relied on matching rsIDs and effect alleles rather than chromosomal coordinates.

#### 2.3.3 MR estimation and sensitivity analyses

Once the exposure and outcome data were harmonized, two-sample MR analyses were implemented through the TwoSampleMR framework in R. Effect estimates for genetically predicted gene or protein expression on AAA were derived primarily using the inverse-variance weighting (IVW) model, which operates under the assumption that directional horizontal pleiotropy does not influence the results (Burgess et al., 2013). Sensitivity analyses were performed using MR-Egger, weighted median, weighted mode, and simple mode estimators to assess the stability of the findings across different model assumptions (Bowden et al., 2015; Bo et al., 2016). Heterogeneity was quantified through Cochran’s Q test, while directional pleiotropy was inferred from the MR-Egger intercept. Leave-one-out analysis and funnel plot inspection further assessed single-instrument influence and small-sample bias.

Disease-related genes were prioritised using a three-step procedure: (i) an exploratory screening threshold of IVW *p* < 0.05 to minimize the premature elimination of biologically relevant signals; (ii) directional concordance of odds ratios (ORs) across multiple MR estimators; and (iii) exclusion of pleiotropic signals as indicated by a significant MR-Egger intercept (*p* < 0.05).

### 2.4 Intersection of MR findings with DEGs

We intersected the DEGs with genes showing significant associations with AAA in the genome-wide MR analyses (eQTL-based and pQTL-based). Overlaps were computed at the gene-symbol level in R using VennDiagram, and the chromosomal distribution of the overlapping sets was visualised with circlize. The resulting intersection sets were considered MR-supported DEGs and were carried forward for downstream analyses.

### 2.5 Feature selection using ML algorithms

The genes subjected to ML analysis were derived from the intersection of eQTL-, pQTL-, and DEG-based results, yielding a refined candidate set relevant across multi-omic datasets. These intersecting genes were subsequently analysed using 3 complementary ML algorithms—least absolute shrinkage and selection operator (LASSO), random forest (RF) and support vector machine-recursive feature elimination (SVM-RFE)—to pinpoint critical genes associated with AAA.

We implemented LASSO regression through the glmnet package for variable selection and regularization, applying the one-standard-error rule to achieve an optimal trade-off between model simplicity and predictive performance (Frost and Amos, 2017). SVM-RFE, performed via the e1071 package with 10-fold cross-validation, recursively discarded low-importance features to improve overall classification performance (Huang et al., 2018). Using the randomForest package, RF analysis quantified gene importance, and genes with importance scores exceeding 2 were identified as key contributors (Hu and Szymczak, 2023). The final gene set was obtained by intersecting the results of the three algorithms, ensuring robust feature selection through cross-method consensus.

### 2.6 MR validation of candidate genes

Focused two-sample MR evaluations were undertaken to examine the genetic relevance of the ML-derived gene set, following the analytic workflow presented in Section 2.3. The IVW approach was used as the main estimator, with MR-Egger, weighted median, and simple mode methods providing sensitivity evaluation. ORs with corresponding 95% confidence intervals (CIs) were estimated to determine both the direction and strength of MR-supported associations. Funnel plots, forest plots, and leave-one-out analyses were applied as sensitivity analyses to evaluate the robustness of the MR effect estimates and potential pleiotropy.

### 2.7 Colocalisation analysis

To strengthen the reliability of the genetic results and detect potential shared underlying variants contributing to AAA, colocalization analyses were conducted on the candidate genes with the coloc R package (Kruschke, 2021). This approach utilised SNPs harmonised via the TwoSampleMR framework, with default prior probabilities set as follows: p_1_ = 1 × 10^−4^ for association with gene expression, p_2_ = 1 × 10^−4^ for association with AAA risk, and p_12_ = 1 × 10^−5^ for association with both traits. Five mutually exclusive scenarios were tested: PPH_0_, indicating absence of association for both traits; PPH_1_, linking effects solely to gene expression; PPH_2_, restricting effects to AAA susceptibility; PPH_3_, implicating two independent underlying variants influencing each trait; and PPH_4_, supporting a shared underlying variant for both traits. A PPH_4_ > 0.80 was regarded as strong evidence of colocalisation. Genes meeting this criterion were considered colocalized candidate genes and potential therapeutic targets, reflecting their likely involvement in both gene regulation and disease pathogenesis.

### 2.8 Expression validation and discriminatory performance

Expression validation was performed for genes that demonstrated strong colocalisation with AAA risk signals (PPH_4_ > 0.80) in the colocalisation analysis. External validation with the GSE7084 dataset involved contrasting the expression profiles of colocalized candidate genes in aneurysmal aortas relative to control tissues. All data handling and statistical testing were performed in R with the limma package, and graphical outputs were generated using ggpubr. ROC curves were generated with the pROC package, and discriminatory accuracy was quantified by calculating the area under the curve (AUC) along with its 95% CIs (Robin et al., 2011). These analyses served to confirm the discriminatory value and cross-cohort reproducibility of the colocalised candidate genes.

### 2.9 GSEA enrichment analysis

To explore the pathways linked to the validated potential biomarker genes, we carried out GSEA using the clusterProfiler package in R (Subramanian et al., 2005). For each gene, the median expression within the AAA samples served as the dividing point for assigning samples to either the high- or low-expression category. Kyoto Encyclopedia of Genes and Genomes (KEGG) pathway analysis was performed via the gseKEGG function with default settings. Genes were ranked by differential expression between the two AAA subgroups, and pathway enrichment was assessed through permutation testing. The leading five enriched pathways per expression group were quantified and visualized, with significance defined as *p* < 0.05.

### 2.10 Immune cell infiltration analysis

The immune microenvironment critically shapes immune cell composition and activity, providing key insights into disease progression and therapeutic responsiveness. We used the single-sample gene set enrichment analysis (ssGSEA) algorithm to profile 28 immune cell subsets, thereby characterizing the immune landscape of AAA (C et al., 2022). Group differences in immune cell infiltration were assessed via the Wilcoxon rank-sum test, with significance defined as *p* < 0.05. We employed Spearman’s rank correlation to quantify associations between immune cell infiltration levels and corresponding gene expression signatures. Correlations achieving a *p* < 0.05 were interpreted as significant, suggesting possible interactions between the candidate genes and immune cell abundance.

### 2.11 Experimental validation of differential gene expression

#### 2.11.1 Murine AAA model

The Animal Ethics Committee of Nanjing First Hospital (DWSY-25083715) approved all animal experimental procedures. All procedures adhered to the National Institutes of Health principles governing laboratory animal use. Eight-week-old SPF male C57BL/6 mice were kept in a regulated environment, with temperature at 25 °C ± 1 °C, humidity at 65% ± 5%, and alternating 12-h light and dark periods. The mice were acclimated to the housing environment for 1 week before AAA induction.

Male mice underwent AAA induction using a calcium chloride (CaCl_2_)-mediated chemical injury model as outlined previously (Li et al., 2024). Mice were randomly assigned to the AAA group or the sham-operated control group (n = 7 per group). Under 4% isoflurane anesthesia, each mouse was laid supine on a controlled heating platform. The operative site was shaved, and a 1.5-cm midline abdominal opening was created to expose the underlying musculature and retroperitoneal structures. Under magnified observation, the abdominal aorta distal to the renal arteries was carefully exposed and freed from surrounding tissues. Aneurysm formation was initiated by applying a gauze pad soaked in 0.5 mol/L CaCl_2_ around the isolated aortic segment for 15 min. Sham-operated controls underwent the same surgical steps but were treated with 0.9% Sodium chloride-soaked gauze. After extensive saline irrigation of the abdominal field, the muscle layer and overlying skin were individually sutured with an interrupted stitching technique. Aortic tissues were harvested 4 weeks after CaCl_2_ or NaCl treatment. Successful AAA induction was confirmed at the time of tissue harvest by macroscopic observation of infrarenal aortic dilation and quantitative measurement of maximum aortic diameter.

#### 2.11.2 Reverse transcription-quantitative polymerase chain reaction (RT-qPCR)

Using the QIAzol extraction kit, total RNA was obtained from murine abdominal aortas. First-strand cDNA synthesis was conducted with oligo primers. Quantitative real-time PCR was then performed using a program consisting of 95 °C for 5 min and 40 subsequent cycles of 95 °C for 1 min, 60 °C for 30 s, and 72 °C for 1 min. Relative mRNA abundance was determined using the 2^-ΔΔCt method. The primers used for mouse PLAU amplification were: forward, GCGCCTTGGTGGTGAAAAAC; reverse, GACACGCATACACCTCCGTT.

#### 2.11.3 Protein expression profiling using Western blot (WB)

Aortic tissues were lysed using a RIPA-based extraction buffer containing inhibitors of proteolytic and phosphatase activity. Protein abundance in the lysates was quantified with a bicinchoninic acid method. After normalization, samples with equivalent protein input were separated on SDS-polyacrylamide gels, followed by electrophoretic transfer onto PVDF membranes. The membranes, after being blocked in 5% non-fat milk, were exposed to primary antibodies directed at PLAU and β-actin and left to incubate overnight at 4 °C. After incubation with HRP-conjugated secondary antibodies, bands were visualized using an enhanced chemiluminescence system. Densitometric analysis was performed in ImageJ, and protein levels were standardized against β-actin.

#### 2.11.4 Enzyme-linked immunosorbent assay (ELISA)

Peripheral blood was collected from mice prior to euthanasia. After coagulation at room temperature for 2 h, blood was centrifuged at 1000 *g* for 20 min to separate the serum, which was then stored at −80 °C until analysis. Circulating PLAU was quantified using a mouse uPA sandwich ELISA kit (Cat. No. MM-0101M2; Meimian, China), following the manufacturer’s protocol. Briefly, the samples and standards were added to a pre-coated microplate and incubated. After washing, a biotin-conjugated detection antibody was added, followed by an enzyme conjugate. The colorimetric reaction was developed using TMB substrate, and the reaction was stopped by adding a stop solution. The optical density was measured immediately at 450 nm using a DR-3518GL microplate reader.

#### 2.11.5 Immunohistochemical analysis

Sections (4 μm) prepared from paraffin-embedded aortic tissue were subjected to standard IHC processing, including deparaffinization, rehydration, and antigen retrieval in citrate buffer (pH 6.0). Endogenous peroxidase was inhibited using 3% hydrogen peroxide, after which the slides were exposed to a PLAU-specific primary antibody and incubated overnight at 4 °C. Visualization was achieved with an HRP-labeled secondary antibody and DAB solution. Following haematoxylin counterstaining, the prepared tissue sections were visualized using a light microscope. Quantitative assessment of PLAU expression was performed by determining integrated optical density values using Image-Pro Plus.

### 2.12 Code availability

The custom R scripts used in this study for MR and ML analyses are available in the GitHub repository: https://github.com/ming-xie-jia/AAA-MR-ML-Analysis.

### 2.13 Statistical analysis

All statistical procedures were implemented with R and GraphPad Prism. For the animal experiment datasets, continuous outcomes were analyzed by Student’s t-test, and values with *p* < 0.05 were interpreted as meaningful.

## 3 Results

### 3.1 PCA and identification of DEGs

**FIGURE 2:**
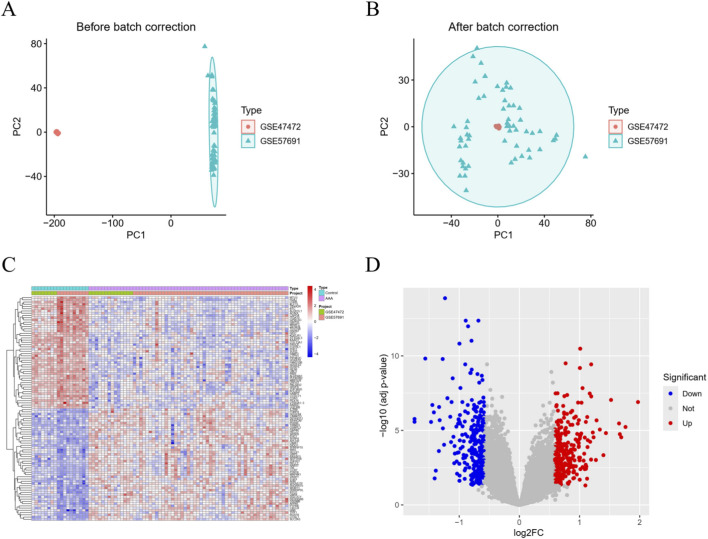
PCA and DEG profiling. **(A,B)** PCA of samples before and after batch correction. **(C)** Heatmap of DEGs between AAA and control tissues. **(D)** Volcano plot illustrating the distribution of DEGs.

We integrated GSE47472 and GSE57691 and applied batch correction to harmonize expression profiles across studies. PCA was conducted to visualise sample clustering patterns before and after batch correction. Prior to correction, the two datasets clustered separately along the first two principal components, revealing substantial batch effects (Figure 2A). After correction, samples from both datasets substantially overlapped (Figure 2B), supporting effective mitigation of batch-related bias and enabling joint analysis.

Normalized data were analyzed using limma to define DEGs, adopting |log2FC| > 0.585 and adj *p*-value <0.05 as cutoff values. A total of 551 DEGs were identified in AAA tissues, of which 277 were upregulated and 274 were downregulated (Supplementary Table S1). DEGs exhibited distinct expression profiles in the heatmap (Figure 2C), and their log2FC and significance distributions were visualized in the volcano plot (Figure 2D).

### 3.2 Disease ontology (DO), gene ontology (GO) and KEGG enrichment of DEGs

**FIGURE 3:**
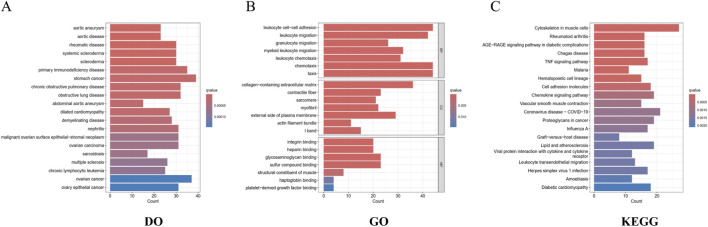
Overview of functional enrichment results for DEGs, including **(A)** DO **(B)** GO and **(C)** KEGG pathway analyses.

DO classification showed that many of the DEGs mapped to disorders involving cardiovascular processes and immune regulation, including aortic aneurysm, aortic disease, rheumatic disease, and chronic obstructive pulmonary disease (Figure 3A; Supplementary Table S2). GO analysis indicated that the DEGs were predominantly enriched in biological processes associated with leukocyte migration, chemotaxis, and cell-cell adhesion; the main cellular components and molecular functions included extracellular-matrix structures, integrin binding, and glycosaminoglycan binding (Figure 3B; Supplementary Table S3). Consistently, analysis of KEGG pathways demonstrated substantial enrichment related to inflammation and vascular-remodeling signatures—such as TNF signaling, AGE-RAGE signaling in diabetic complications, vascular smooth muscle contraction, and cell-adhesion molecules (Figure 3C; Supplementary Table S4).

### 3.3 Genome-wide MR leveraging eQTL and pQTL instruments

Two-sample MR analyses incorporating eQTL- and pQTL-based instruments were conducted to evaluate how gene and protein expression are genetically associated with AAA susceptibility. The IVW framework was adopted as the principal estimation method, with significance defined at *p* < 0.05. To enhance robustness, we retained only exposures showing directionally concordant ORs (all >1 or all <1) across five MR estimators, and we excluded associations with evidence of horizontal pleiotropy (*p* < 0.05). Under these criteria, we identified 267 AAA-associated genes from eQTL-derived instruments and 129 AAA-associated proteins from pQTL-derived instruments (Supplementary Tables S5,S6).

### 3.4 Integration of MR and DEGs

**FIGURE 4:**
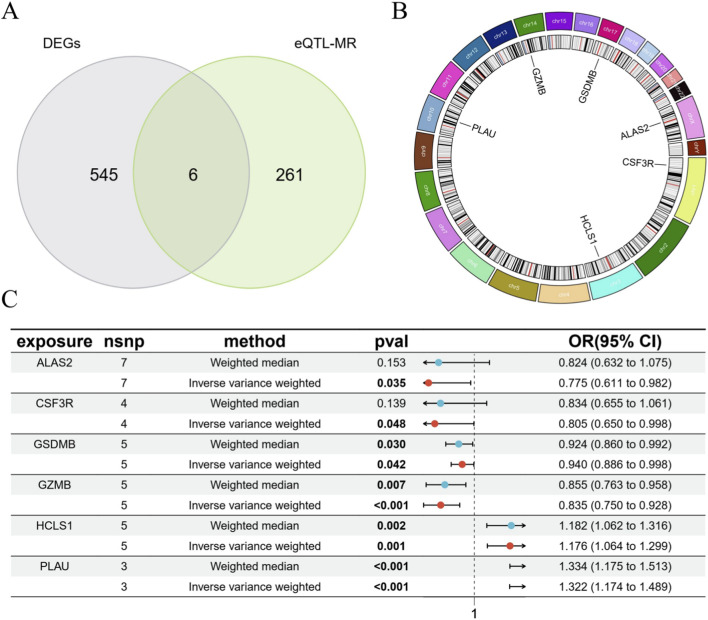
MR-supported genes shared by DEGs and eQTL-based analysis. **(A)** Venn diagram illustrating gene overlap between DEGs and eQTL-MR-derived genes. **(B)** Circos plot displaying the chromosomal locations of the 6 overlapping genes. **(C)** Forest plot summarizing MR estimates for the 6 candidate genes.

**FIGURE 5:**
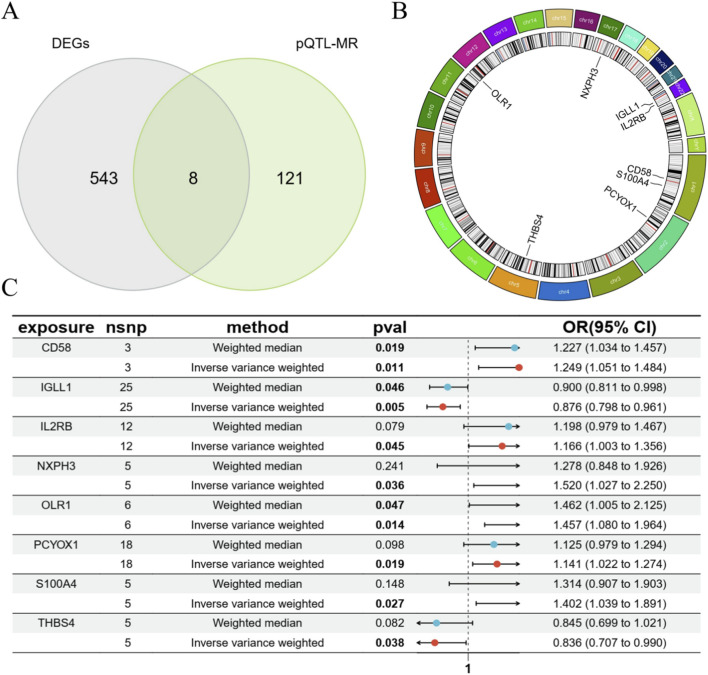
MR-supported genes shared by DEGs and pQTL-based analysis. **(A)** Venn diagram illustrating gene overlap between DEGs and pQTL-MR-derived genes. **(B)** Circos plot displaying the chromosomal locations of the 8 overlapping genes. **(C)** Forest plot summarizing MR estimates for the 8 candidate genes.

We intersected the DEGs with the genome-wide MR findings derived from eQTL- and pQTL-based instruments. As shown in Figure 4A, 6 genes were found to overlap with both the DEGs and the eQTL-based MR-supported gene set; in parallel, 8 genes intersecting with the pQTL-derived MR-supported set were identified (Figure 5A). The chromosomal distribution of these candidates is displayed in Circos plots (Figures 4B, 5B). Targeted MR estimates for each overlapping gene are summarized as forest plots (Figures 4C, 5C), with IVW results used for significance calling (*p* < 0.05). ORs <1 were interpreted as lower risk, whereas ORs >1 were interpreted as higher risk. Across the combined eQTL- and pQTL-based MR analyses, 6 genes were classified as putatively protective (OR < 1) and 8 as risk-associated (OR > 1).

### 3.5 ML-based feature selection

**FIGURE 6:**
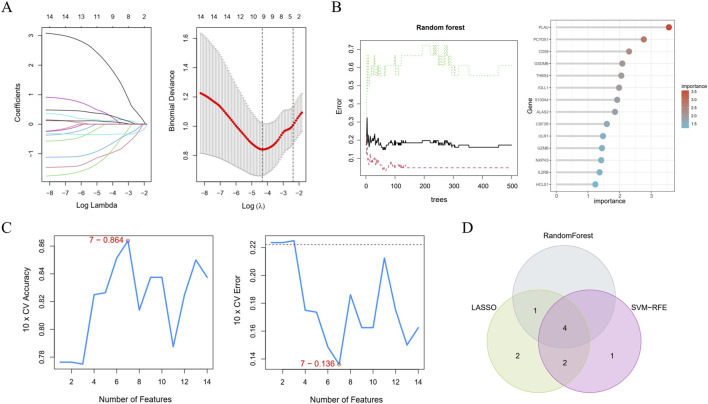
ML-based feature selection of candidate genes. **(A)** LASSO regression for feature selection. **(B)** RF model and variable importance ranking. **(C)** SVM-RFE feature selection based on cross-validation performance. **(D)** Venn diagram showing the 4 genes consistently selected by all three algorithms.

Three ML methods (LASSO, RF, and SVM-RFE) were employed to conduct an additional round of feature refinement for the 14-gene candidate set. LASSO, with the penalty parameter chosen at the minimum cross-validated error, retained 9 genes (PLAU, GSDMB, CSF3R, IGLL1, CD58, OLR1, PCYOX1, S100A4, THBS4; Figure 6A). RF identified 5 genes with relative importance >2 (PLAU, PCYOX1, CD58, GSDMB, THBS4; Figure 6B). SVM-RFE, optimized at the minimum cross-validated error, selected 7 genes (PLAU, OLR1, PCYOX1, CD58, ALAS2, THBS4, CSF3R; Figure 6C). Intersecting the outputs of the three algorithms yielded 4 ML-prioritized genes: PLAU, CD58, PCYOX1, and THBS4 (Figure 6D).

### 3.6 MR analysis of candidate genes

We conducted targeted two-sample MR to investigate the genetic relevance of 4 ML-prioritised genes—PLAU, CD58, PCYOX1, and THBS4—in AAA. Based on the IVW model as the primary MR estimator, all 4 ML-derived genes demonstrated significant MR-supported associations with AAA risk, including PLAU (OR = 1.322, 95% CI: 1.174–1.489, *p* < 0.001), CD58 (OR = 1.249, 95% CI: 1.051–1.484, *p* = 0.011), PCYOX1 (OR = 1.141, 95% CI: 1.022–1.274, *p* = 0.019), and THBS4 (OR = 0.836, 95% CI: 0.707–0.990, *p* = 0.038).

**FIGURE 7:**
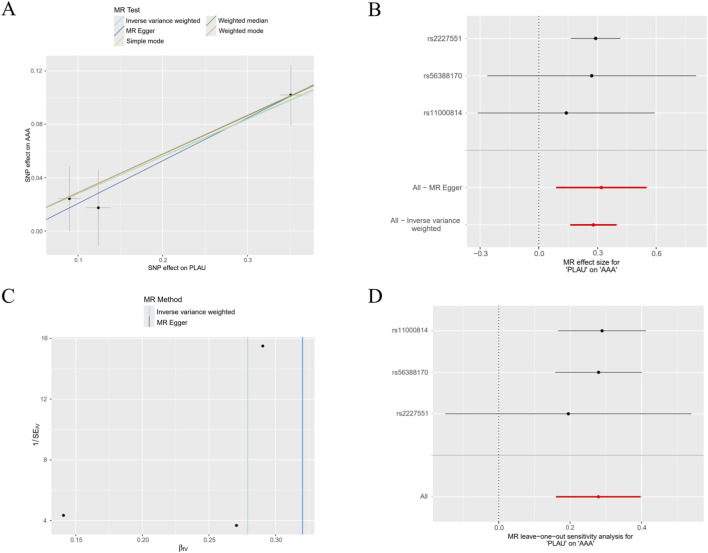
MR analysis of the association between PLAU expression and AAA. **(A)** Scatter plot of SNP-level effects across five MR methods. **(B)** Forest plot of individual SNP estimates. **(C)** Funnel plot assessing heterogeneity. **(D)** Leave-one-out analysis confirming result robustness.

The full MR panels for PLAU are shown in Figure 7, and corresponding results for CD58, PCYOX1, and THBS4 are provided in Supplementary Tables S1–S3. Scatter plots depict SNP-level effects and pooled MR effect estimates derived from multiple MR methods (Figure 7A; Supplementary Figures S1A–S3A). SNP-level forest plots (Figure 7B; Supplementary Figures S1B–S3B) indicate directionally concordant associations across IVs. Detailed information on the instrumental SNPs used for each candidate gene is provided in Supplementary Table S7. Funnel plots were largely symmetric (Figure 7C; Supplementary Figures S1C–S3C), and Cochran’s Q statistics revealed no significant heterogeneity among the IVs (*p* > 0.05; Supplementary Table S8). In addition, MR-Egger intercept tests indicated no significant directional pleiotropy (*p* > 0.05, Supplementary Table S9). Leave-one-out analyses further demonstrated that the MR effect estimates were not driven by any single SNP (Figure 7D; Supplementary Figures S1D–S3D), confirming the robustness and stability of the MR findings.

### 3.7 Colocalization analysis

**FIGURE 8:**
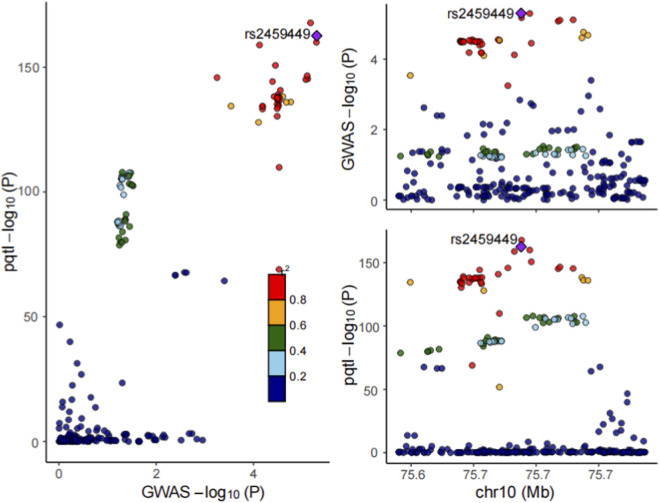
Colocalization analysis of the candidate genes. Colocalization plot for PLAU (PPH4 = 0.983), indicating strong evidence that a shared variant influences both PLAU expression and AAA risk. Dot colors represent LD (*r* ^2^) with the lead SNP rs2459449.

Colocalization analysis was performed using the coloc framework to assess whether the associations between gene expression and AAA risk were attributable to a shared variant across the 4 candidate genes. PLAU exhibited strong colocalization evidence, with a posterior probability for a shared variant (PPH4 = 0.983), implying a shared genetic association signal between PLAU expression and AAA risk (Figure 8). In contrast, CD58, PCYOX1, and THBS4 provided little support for colocalization, with PPH4 values far below the predefined threshold for shared variants (PPH4 > 0.80) (Supplementary Table S10; Supplementary Figures S4–S6). This suggests that the expression signals for these genes and AAA risk loci are unlikely to be explained by the same underlying variant, and may instead be driven by LD with nearby causal variants.

### 3.8 Expression validation and discriminatory performance of PLAU

**FIGURE 9:**
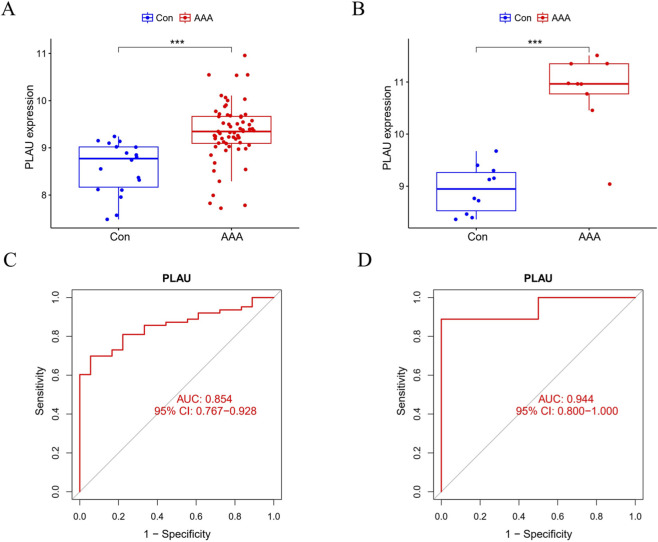
Validation of PLAU expression and discriminatory performance in AAA. **(A)** PLAU expression levels in AAA versus control tissues in the training cohort. **(B)** PLAU expression levels in AAA versus control tissues in the validation cohort. **(C)** ROC curve for PLAU in the training cohort. **(D)** ROC curve for PLAU in the validation cohort. ^***^ *p* < 0.001.

To evaluate the clinical relevance of PLAU, we examined its expression in AAA versus control tissues across two independent cohorts. PLAU expression was markedly elevated in AAA compared with controls in both datasets (*p* < 0.001, Figures 9A,B). We next assessed discriminatory performance using ROC analysis. In the initial dataset, PLAU displayed an AUC of 0.854 (95% CI: 0.767–0.928), whereas assessment in an independent cohort resulted in an AUC of 0.944 (95% CI: 0.800–1.000) (Figures 9C,D). These findings underscore its high discriminatory precision and consistent performance.

### 3.9 GSEA and immune infiltration in AAA

**FIGURE 10:**
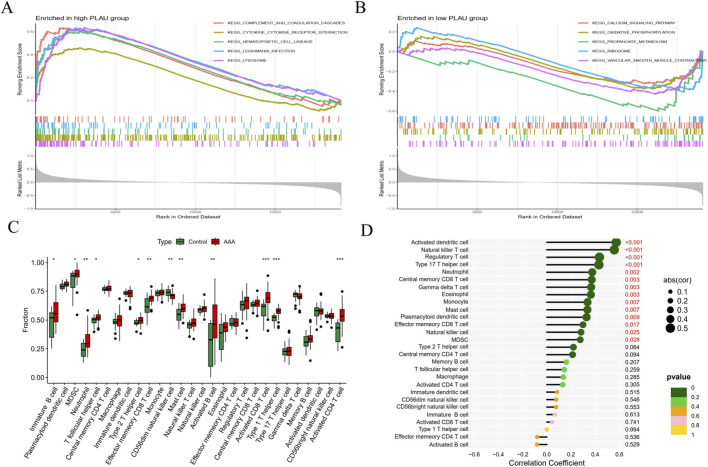
GSEA and immune infiltration profiling of PLAU in AAA. **(A,B)** Enriched pathways identified by GSEA in high- and low-PLAU expression groups within AAA samples. **(C)** Violin plot displaying the distribution of infiltrating immune cells. **(D)** Lollipop plot visualizing the link between PLAU expression and levels of immune cell infiltration.

To elucidate potential biological processes, we performed GSEA of KEGG pathways within the AAA samples of the training cohort after stratifying them by PLAU expression (Figures 10A,B). High-PLAU expression was linked to enrichment of immune-related and inflammatory pathways—including complement and coagulation cascades, cytokine-cytokine receptor interaction, hematopoietic cell lineage, Leishmania infection, and lysosome. In contrast, the low-PLAU group was enriched for metabolism, protein synthesis, and vascular regulation, including calcium signaling pathway, oxidative phosphorylation, propanoate metabolism, ribosome, and vascular smooth muscle contraction. In parallel, ssGSEA detected differential infiltration of 12 out of 28 immune cell subsets between AAA and controls, highlighting substantial remodeling of the immune microenvironment (Figure 10C). Furthermore, analysis of correlations showed that PLAU expression was strongly linked to multiple immune subsets (Figure 10D)—most notably activated dendritic cells (DCs), natural killer T (NKT) cells, regulatory T cells (Tregs), and T helper 17 (Th17) cells—whereas no significant association was observed with memory B cells, effector memory CD4^+^ T cells, or activated CD8^+^ T cells (*p* > 0.05).

### 3.10 Validation in a murine AAA model

**FIGURE 11:**
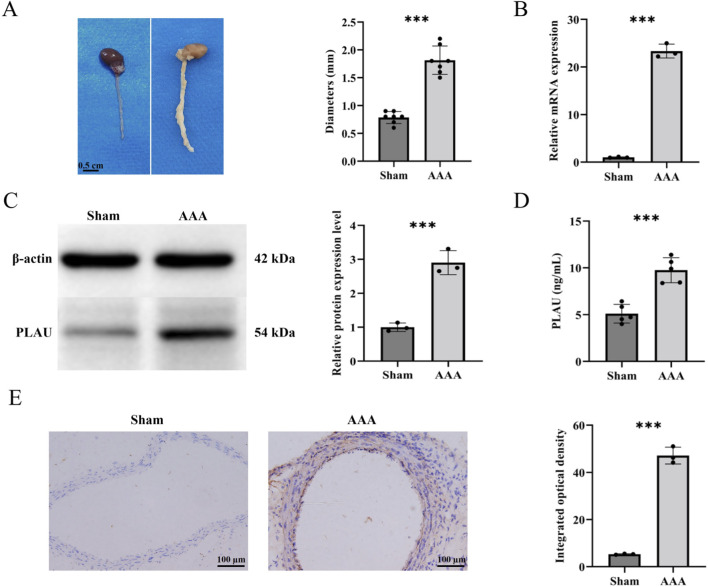
Evaluation of PLAU expression in a murine model of AAA. **(A)** Aortic morphology and diameter in AAA and sham mice (n = 7 per group). **(B)** qRT-PCR analysis of PLAU mRNA in aortic tissues (n = 3 per group). **(C)** WB validation of PLAU protein levels (n = 3 per group). **(D)** ELISA analysis of circulating PLAU levels (n = 5 per group). **(E)** Immunohistochemical detection of PLAU in aortas from AAA and sham groups (n = 3 per group). ^***^ *p* < 0.001.

We further evaluated PLAU expression in a murine AAA model. Gross inspection revealed evident aortic enlargement in AAA mice, and quantitative measurements confirmed a significant increase in external aortic diameter compared with controls (Figure 11A), indicating successful model induction. Consistent with this phenotype, RT-qPCR showed higher PLAU mRNA levels in aortic tissue from AAA mice than from sham controls (Figure 11B). Concordantly, WB analysis demonstrated a significant 2.9-fold elevation in PLAU protein abundance in AAA tissue compared to sham controls (Figure 11C; Supplementary Figure S7). Furthermore, ELISA analysis showed a significant elevation of circulating PLAU levels in the peripheral blood of AAA mice (Figure 11D). Moreover, immunohistochemistry revealed stronger PLAU immunoreactivity in AAA aortas, consistent with the molecular assays (Figure 11E).

## 4 Discussion

AAA imposes a substantial clinical burden because it often progresses silently until catastrophic rupture. Although imaging enables detection at more advanced stages, the absence of robust molecular biomarkers limits early diagnosis and timely intervention (Golledge et al., 2023). To move beyond correlation-based findings that dominate prior transcriptomic studies, we applied an integrative multi-omics framework that couples MR with ML-driven prioritization. This design provides genetic anchoring for candidate biomarkers while maintaining predictive rigor, offering a more biologically interpretable alternative to conventional approaches (Zhu et al., 2024).

To delineate transcriptional alterations in AAA, we analyzed two independent microarray datasets (GSE47472 and GSE57691) and identified 551 DEGs. Gene-set enrichment implicated immune activation, ECM remodeling, leukocyte chemotaxis, and vascular inflammation, aligning with established features of AAA pathobiology (Li and Kong, 2020). However, the control samples in these datasets primarily consist of organ donors and autopsy specimens, which may introduce general atherosclerosis markers alongside AAA-specific changes. Furthermore, batch correction across datasets may inadvertently mask true biological heterogeneity (Leek et al., 2010). To overcome such combined biological and technical confounding, and move beyond mere association, we integrated AAA GWAS summary statistics with eQTL and pQTL resources to prioritize genes whose expression or circulating protein levels may genetically influence disease risk. By leveraging germline variants as IVs, MR minimized confounding and reverse causation, leading to the identification of 267 putative AAA-related genes from eQTL and 129 proteins from pQTL data. We deliberately employed an exploratory MR screening threshold (*p* < 0.05) at this initial stage to cast a wide net, avoiding the premature elimination of biologically relevant signals. While this permissive threshold inherently introduces a false-positive burden, our subsequent multi-omics intersection and rigorous experimental validation were specifically designed to mitigate this risk.

Complex diseases typically emerge from multilayered molecular interactions spanning the genome, transcriptome, and proteome. Multi-omics designs capture these complementary dimensions and yield a more integrated view of disease biology (Doran et al., 2021). In this study, transcriptomic profiling identified 551 DEGs associated with AAA. To appraise their genetic relevance and pathophysiological context, we integrated AAA GWAS summary statistics with eQTL and pQTL. Intersecting MR-supported signals with the DEG set yielded 6 overlapping genes on the eQTL axis and 8 on the pQTL axis—high-priority candidates that couple genetic evidence with transcriptional dysregulation. This integrative strategy aggregates pathophysiological information across molecular layers and facilitates the nomination of robust biomarker candidates for AAA.

Although AAA is anatomically localized to the aorta, its pathogenesis is closely linked to systemic immune-inflammatory responses (Yuan et al., 2020). Because circulating leukocytes are a major source of immune cells recruited into the aneurysmal wall, whole-blood-derived eQTLs and plasma-derived pQTLs may serve as biologically plausible proxies for capturing part of this systemic inflammatory background (Raffort et al., 2017). In this context, integrating large-scale circulation-derived QTL resources provides a biologically informative framework for prioritizing AAA-related biomarker candidates.

Recent advances in ML have improved the analysis of complex genetic and transcriptomic data by identifying subtle, high-dimensional patterns (Toh et al., 2019). To effectively handle inherent multicollinearity and prevent small-sample overfitting, we implemented an ensemble machine-learning pipeline (LASSO, SVM-RFE, and RF) rather than relying on simple statistical prioritization (Huang et al., 2025). By applying this strategy to refine the MR- and transcriptomics-derived candidate set, we consistently prioritized 4 genes: PLAU, CD58, PCYOX1, and THBS4. As previous research has suggested, significant MR findings can sometimes be driven by SNPs in LD, leading to false-positive outcomes (Yan et al., 2025). To assess their genetic relevance and rule out potential confounding by LD, we employed Bayesian colocalization analysis. Our results revealed that only PLAU exhibited strong support for a shared genetic association signal with AAA risk loci (PPH4 = 0.983), suggesting it as a highly plausible candidate at this locus. In contrast, the other 3 genes showed no such evidence, suggesting their initial MR signals were likely confounded by LD. Notably, targets supported by both MR and colocalization are empirically more likely to succeed in drug development, underscoring the translational promise of PLAU (Zheng et al., 2020). Across the training and independent validation cohorts, PLAU was consistently upregulated in AAA and achieved robust discriminatory performance (AUCs >0.85). Its elevation was corroborated in a CaCl_2_-induced AAA mouse model at both mRNA and protein levels. Importantly, ELISA confirmed that this local upregulation is accompanied by significantly elevated circulating PLAU levels in the peripheral blood. Furthermore, immunohistochemistry localized PLAU predominantly to the medial and adventitial layers—regions consistent with active pathological remodeling.

PLAU encodes uPA, a serine protease that mediates the transformation of plasminogen into plasmin, subsequently enhancing fibrinolytic activity and driving ECM remodeling (Li et al., 2021). Beyond mediating proteolysis, the uPA system contributes to chemotactic responses, cellular adhesion and migration, the promotion of angiogenesis, and the regulation of inflammatory pathways (Hamada et al., 2024). Dysregulated PLAU expression is reported across several malignancies, where it promotes invasion and metastasis through ECM remodeling and activation of latent growth factors (Mahmood et al., 2018). Within the cardiovascular system, accumulating evidence associates elevated PLAU/uPA activity with endothelial dysfunction, atherosclerotic-plaque instability, and increased risk of coronary occlusive events (Song et al., 2022). Consistently, transcriptomic studies highlight PLAU as a putative marker of vascular inflammation and disease progression in cardiovascular disorders (Ukkat et al., 2023).

Despite extensive evidence linking PLAU to diverse pathological processes, its specific contribution to AAA remains incompletely defined. In line with evidence that immune-driven inflammation is pivotal in AAA development. GSEA demonstrated pronounced activation of immune-inflammatory pathways in AAA samples with high PLAU expression, such as complement and coagulation cascades, cytokine-receptor interactions, hematopoietic cell lineage, and lysosome-associated signalling. These pathways coordinate leukocyte activation, cytokine signaling, and immune-cell differentiation—functions integral to aneurysmal wall remodeling (Li and Kong, 2020). In parallel, MR indicated that elevated PLAU expression confers a higher susceptibility to AAA (OR > 1), providing genetic support that complements the transcriptional enrichment and argues for a biologically coherent, inflammation-centered pattern. This immune-centered pattern is consistent with a systemic-to-local inflammatory link in AAA.

Immune-cell infiltration analysis further revealed positive correlations between PLAU expression and several activated immune subsets—notably activated DCs, NKT cells, and Th17 cells—which promote antigen presentation, effector priming, and vascular inflammation (Miao et al., 2021; Liu et al., 2019). Correlations with Tregs should be interpreted cautiously: although Tregs typically exert immunoregulatory or protective effects, their increase in inflamed tissue can reflect compensatory recruitment or context-dependent plasticity rather than a pro-inflammatory role (Ait-Oufella and Tedgui, 2016). These patterns align with reports that PLAU is associated with immune-cell infiltration and reflects changes in the immune landscape across malignant and chronic inflammatory diseases, underscoring its potential as an immunomodulatory node (Hindy et al., 2022). By contrast, PLAU showed no material association with memory B cells or effector memory CD4^+^ T cells, suggesting cell-type specificity. Moreover, the low-PLAU group was enriched for oxidative phosphorylation, ribosomal, and calcium-signaling pathways, compatible with a more quiescent vascular state. Collectively, the evidence suggests that increased PLAU expression correlates with immune activation and vascular inflammatory responses, supporting its relevance as a molecular signature associated with AAA progression. While our bulk transcriptomic-based findings are exploratory, future studies integrating single-cell sequencing and circulating cytokines are required to fully validate the systemic PLAU-immune network and its role in immune inflammation activation in AAA.

This study has several limitations. First, all transcriptomic data were obtained from GEO microarray cohorts, which are subject to inter-study variation and batch effects; thus, residual confounding cannot be fully excluded despite batch correction and external validation. Particularly, this includes the inherent biological heterogeneity introduced by integrating distinct anatomical regions. Second, the reliance of the MR analyses on predominantly European-ancestry datasets may limit their transferability to other populations, highlighting the necessity for verification in ethnically broader cohorts, including Asian populations. Third, because the primary genetic resources used in this study were derived from the circulation, the present framework may provide only a partial view of vascular-wall-specific regulation in AAA. Fourth, the lack of direct serum or plasma validation in large-scale human cohorts limits the immediate translation of PLAU into a clinical diagnostic blood test, making it a necessary focus for future investigations. Finally, although our colocalization analysis indicates a shared association signal between PLAU expression and AAA risk, it does not establish PLAU as the sole functional mediator at this locus. Moreover, the mechanistic role of PLAU in AAA development remains unclear, and further functional studies, including PLAU perturbation, are needed to clarify its molecular pathways and therapeutic relevance.

## 5 Conclusion

Integrating genetic associations, proteomic evidence, and experimental validation, we identify PLAU as a genetically supported candidate gene with strong *in vivo* expression concordance in AAA. The convergence of MR, ML, and colocalization underscores its robustness across analytic layers. Furthermore, transcriptomic profiling associates PLAU with immune-inflammatory remodeling in AAA. These findings not only provide a biological context for precision biomarker development but also exemplify a scalable paradigm for multi-omics-driven discovery in complex vascular disorders.

## Data Availability

The primary findings of this study, together with detailed supporting information, are presented in the main manuscript and the Supplementary Materials. Any requests for clarification may be directed to the corresponding authors.
