# Supplementary material for: Multi-omics and machine learning-based exploration of key genes associated with abdominal aortic aneurysm
Source: Front Mol Biosci. 2026 May 29;13:1786983. doi: 10.3389/fmolb.2026.1786983 (PMC13259990; doi:10.3389/fmolb.2026.1786983)
Supplement: Supplementary file 3 [file Supplementaryfile1.docx]

Supplementary Figure S1:


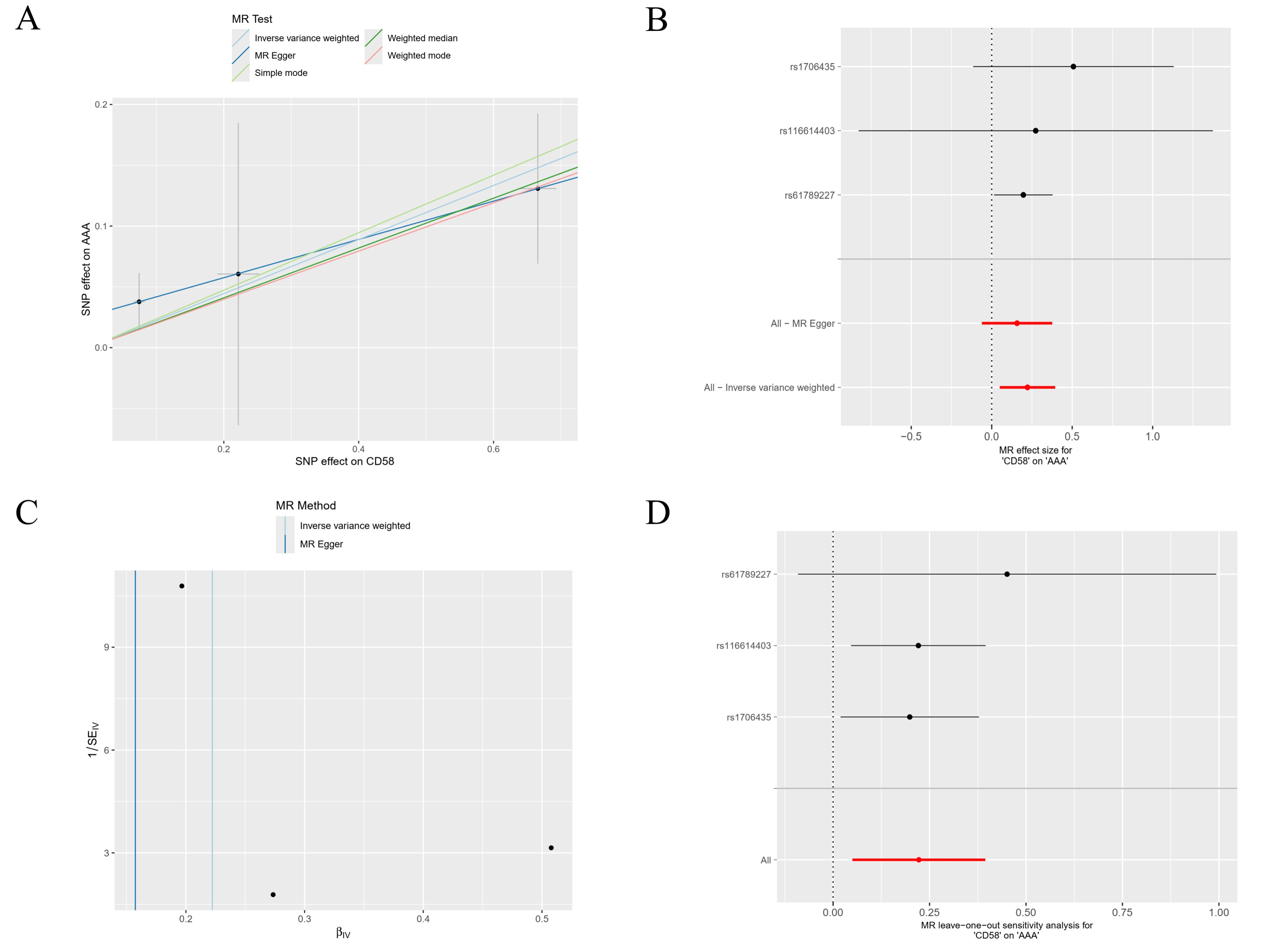


**Fig. S1** MR analysis for the causal association between CD58 expression and AAA. (A) Scatter plot of SNP-level effects across 5 MR methods. (B) Forest plot of individual SNP estimates. (C) Funnel plot assessing heterogeneity. (D) Leave-one-out analysis confirming result robustness.

Supplementary Figure S2:


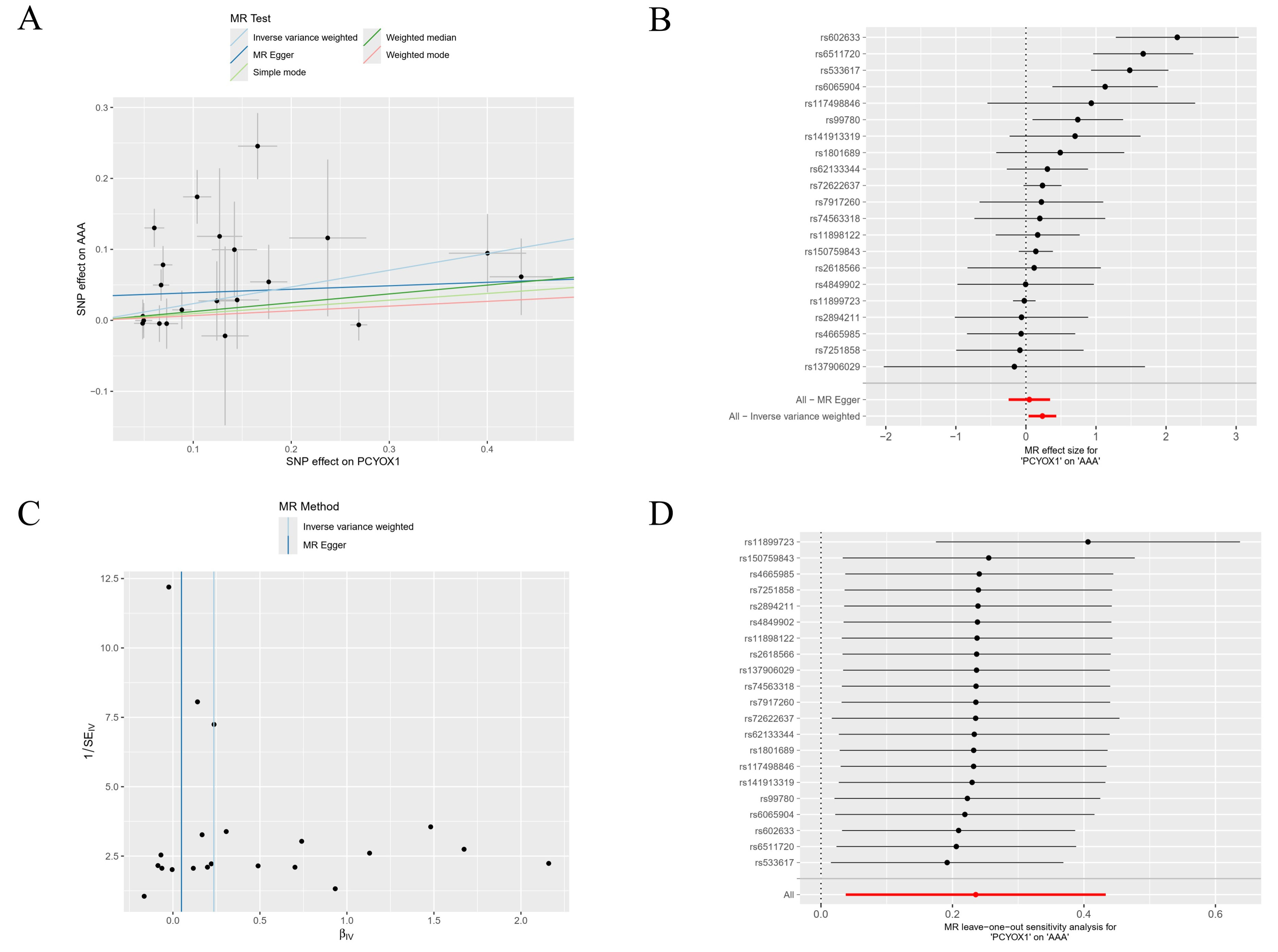


**Fig. S2** MR analysis for the causal association between PCYOX1 expression and AAA. (A) Scatter plot of SNP-level effects across 5 MR methods. (B) Forest plot of individual SNP estimates. (C) Funnel plot assessing heterogeneity. (D) Leave-one-out analysis confirming result robustness.

Supplementary Figure S3:


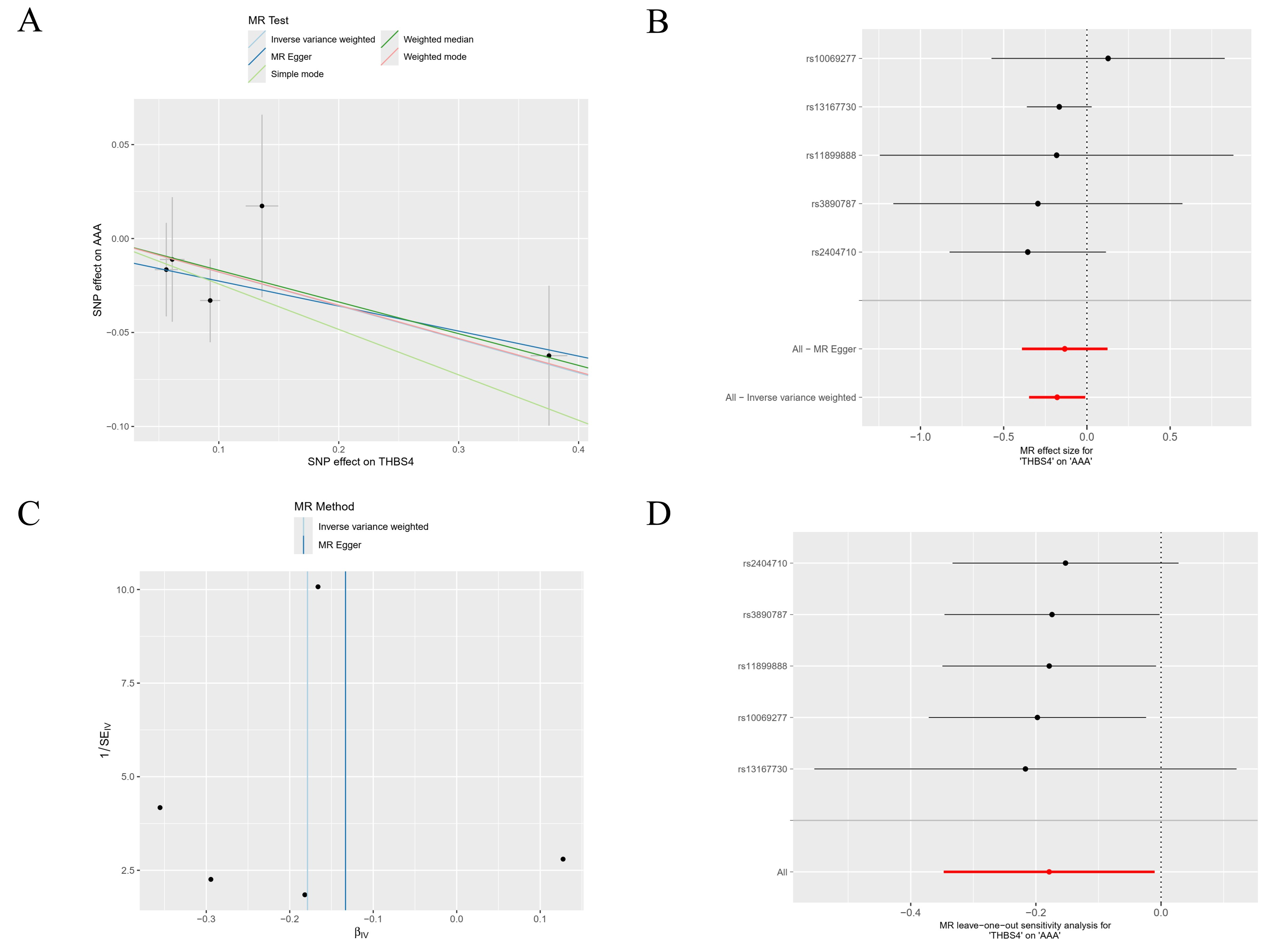


**Fig. S3** MR analysis for the causal association between THBS4 expression and AAA. (A) Scatter plot of SNP-level effects across 5 MR methods. (B) Forest plot of individual SNP estimates. (C) Funnel plot assessing heterogeneity. (D) Leave-one-out analysis confirming result robustness.

Supplementary Figure S4:


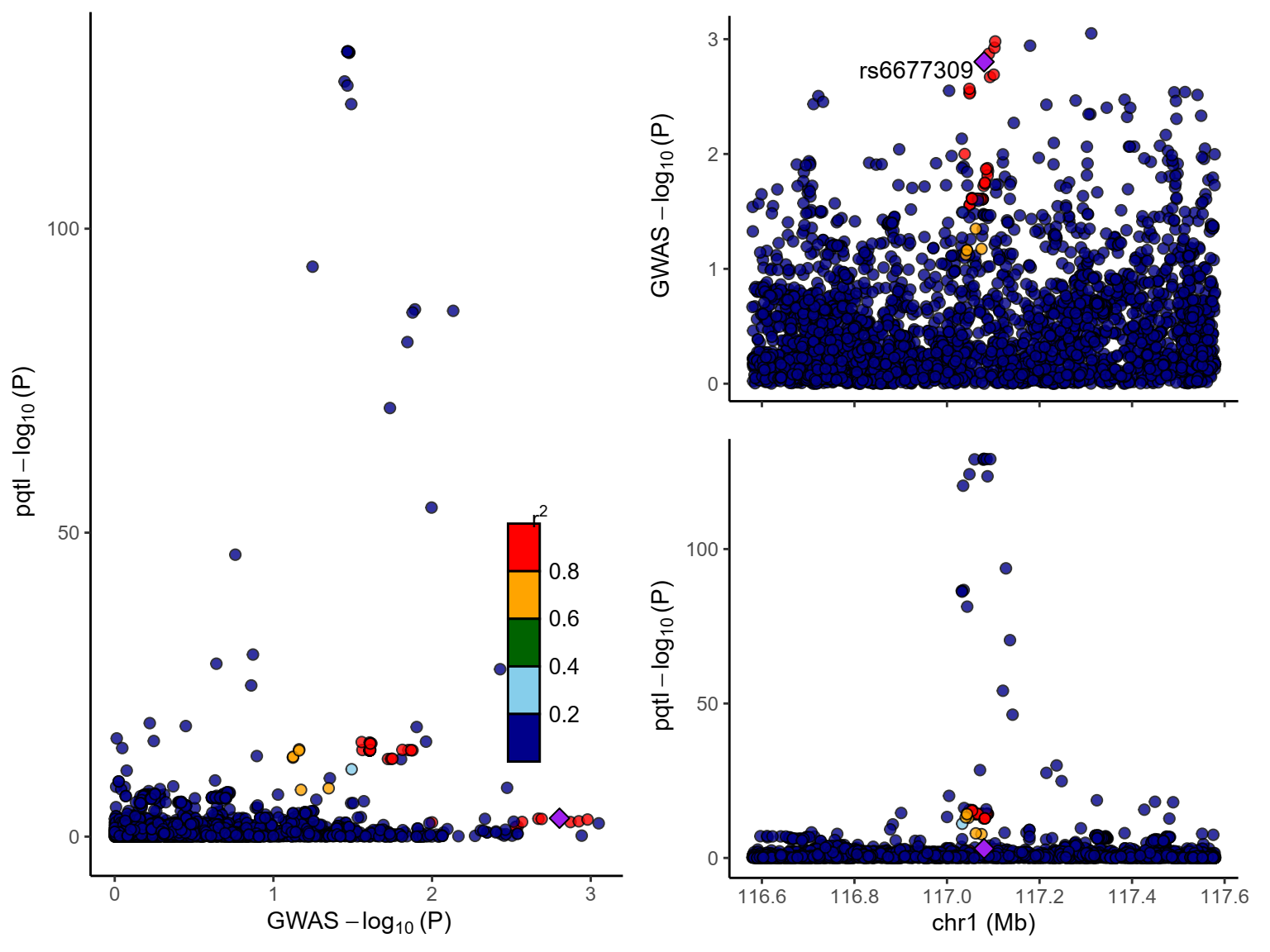


**Fig. S4** Colocalization analysis of CD58. Regional association plots show limited evidence of colocalization between CD58 expression and AAA risk (PPH4 < 0.08). Dot colors indicate LD (r²) with the lead SNP rs6677309.

Supplementary Figure S5:


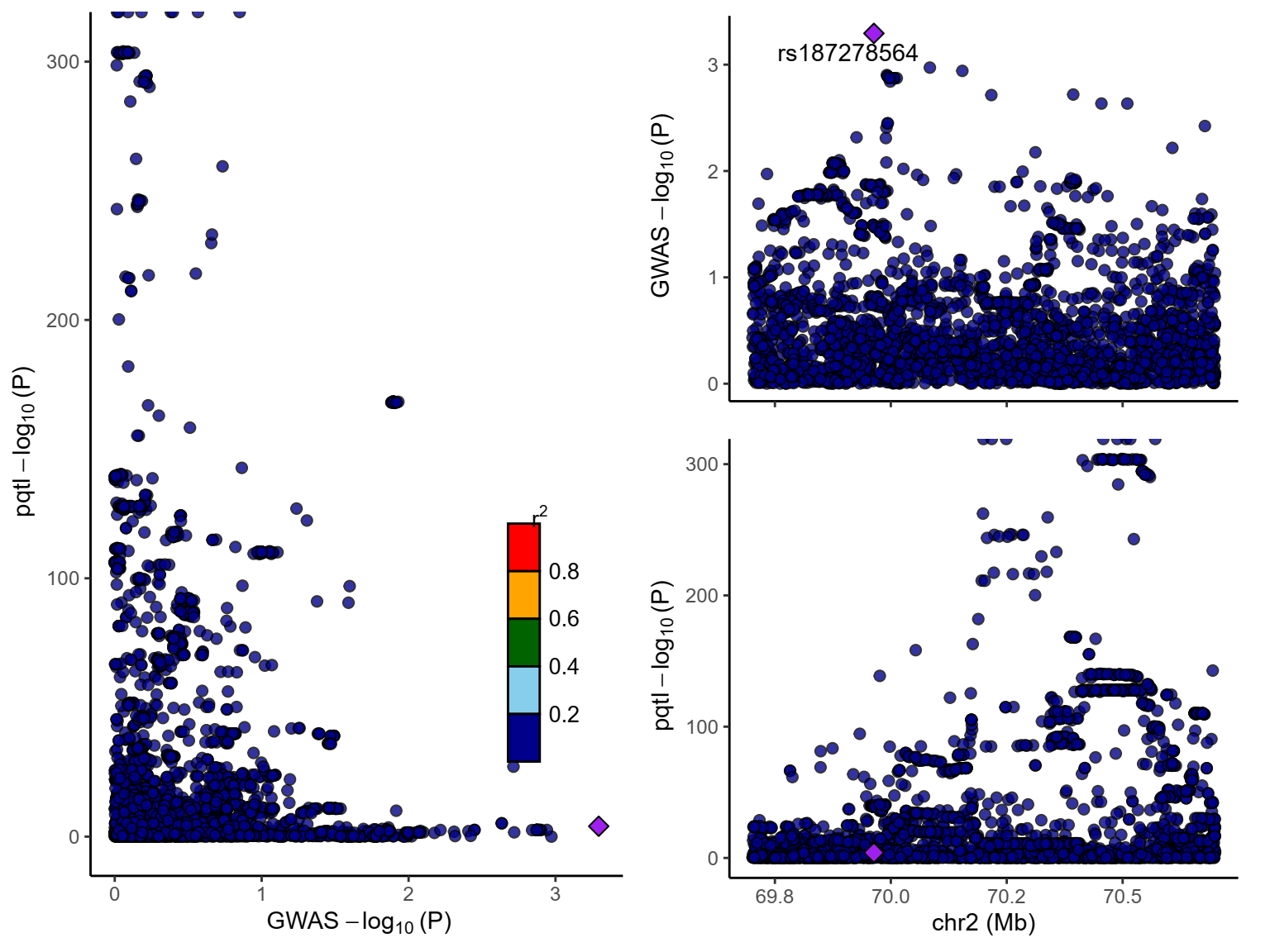


**Fig. S5** Regional association plots show limited evidence of colocalization between PCYOX1 expression and AAA risk (PPH4 < 0.08). Dot colors indicate LD (r²) with the lead SNP rs187278564.

Supplementary Figure S6:


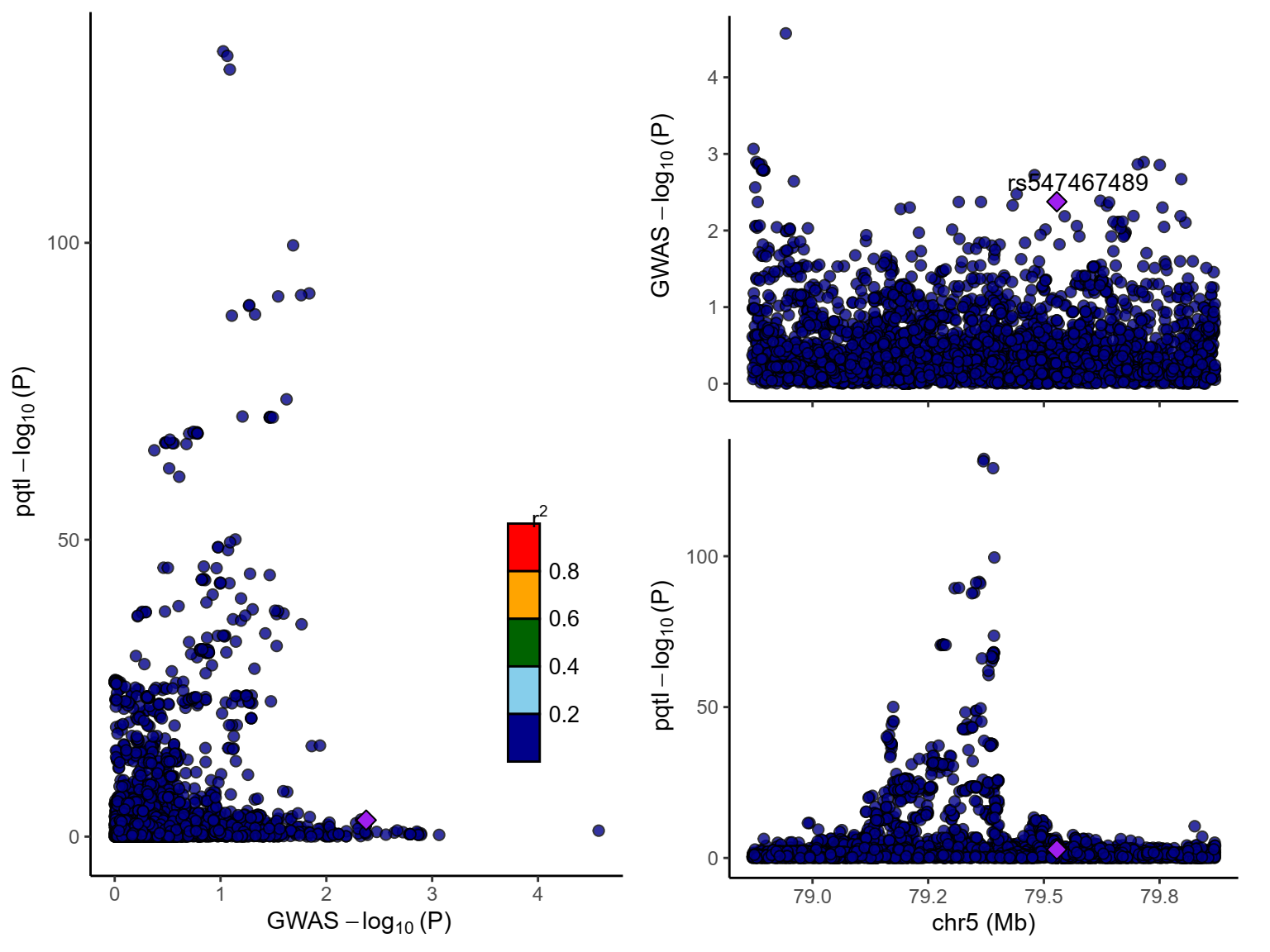


**Fig. S6** Regional association plots show limited evidence of colocalization between THBS4 expression and AAA risk (PPH4 < 0.08). Dot colors indicate LD (r²) with the lead SNP rs547467489.

Supplementary Figure S7:


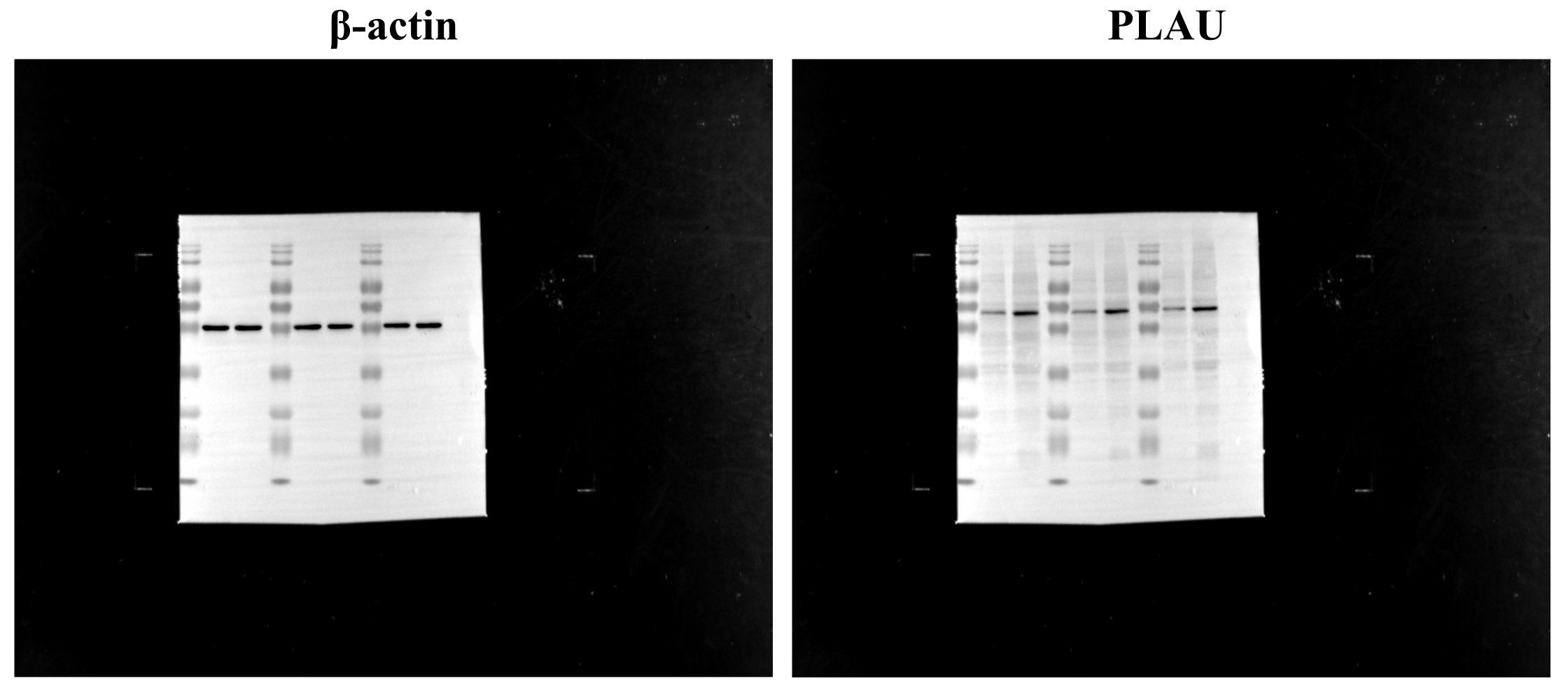


**Fig. S7** Full, uncropped Western blot images of β-actin and PLAU.
